# Parents face quantity–quality trade-offs between reproduction and investment in offspring in Iceland

**DOI:** 10.1098/rsos.160087

**Published:** 2016-05-18

**Authors:** Robert Francis Lynch

**Affiliations:** Department of Anthropology, University of Missouri, Columbia, MO 65201, USA

**Keywords:** life-history traits, lifetime reproductive success, quantity–quality trade-off, parental investment, heritability

## Abstract

How to optimally allocate time, energy and investment in an effort to maximize one's reproductive success is a fundamental problem faced by all organisms. This effort is complicated when the production of each additional offspring dilutes the total resources available for parental investment. Although a quantity–quality trade-off between producing and investing in offspring has long been assumed in evolutionary biology, testing it directly in humans is difficult, partly owing to the long generation time of our species. Using data from an Icelandic genealogy (Íslendingabók) over two centuries, I address this issue and analyse the quantity–quality trade-off in humans. I demonstrate that the primary impact of parents on the fitness of their children is the result of resources and or investment, but not genes. This effect changes significantly across time, in response to environmental conditions. Overall, increasing reproduction has negative fitness consequences on offspring, such that each additional sibling reduces an individual's average lifespan and lifetime reproductive success. This analysis provides insights into the evolutionary conflict between producing and investing in children while also shedding light on some of the causes of the demographic transition.

## 1. Background

Life-history theory provides evolutionary biologists with a framework for understanding how organisms distribute investment in survival, growth and reproduction [1–3]. Research has revealed a negative relationship between growth rate and both lifespan and body size [4–8]. Parents may therefore face a trade-off between reproduction and parental care (or a ‘quantity–quality’ trade-off). This principle is now a basic tenet in evolutionary biology [9–12], and it relies on four assumptions: (i) resources are limited; (ii) as investment in offspring number increases, investment per offspring decreases; (iii) parental investment (PI) [13] in offspring increases offspring's fitness; and (iv) parental fitness depends on the number of offspring that parents successfully raise to sexual maturity and, ultimately, on the number of grandchildren produced [7].

To date, some of the best evidence for the quantity–quality trade-off has come from research on birds [14,15]. Lack [9] pioneered this research by experimentally manipulating clutch sizes and showing that female gulls naturally produce the optimal number of eggs which maximizes their grand-offspring. The fitness effects of balancing parental care and reproduction are often more subtle, however, and most studies of quantity–quality effects have shown either mixed or more complex results [16–18]. One reason that detecting trade-offs can be complicated is because optimal investment strategies often change across time as they respond to shifting environmental conditions [7,19].

Nevertheless, life-history theory has identified a number of environmental factors that often have important effects on reproductive strategies, including environmental quality, environmental predictability and degree of competition [7]. These factors interact in complex ways. For instance, in less predictable environments, individuals who produce more offspring, and subsequently invest less in each, may be at an advantage owing to the increased chances that at least one will survive to reproduce. More predictable environments, however, may generate selection for the production of fewer, higher-quality offspring. Meanwhile, in more predictable environments that also have high adult and juvenile mortality rates, organisms are expected to invest more in offspring quantity and less in quality [19].

Volatile and predictable environments can generate similar results, which often depend upon risk of mortality [20]. For instance, in environments with both low adult and juvenile mortality, parents may be better off either investing in their own growth and survival by delaying reproduction, or by investing more in current offspring (e.g. by increasing birth intervals) [21]. Evidence that individuals often delay the age at which they first give birth in low mortality environments provides support for this hypothesis [22]. At the same time, investing more in quantity has been shown to be adaptive in environments with high child mortality [23]. Optimal reproductive and investment strategies ultimately depend on the pay-offs for producing and investing in children in the face of uncertain environments. Overall, there is a considerable amount of theoretical [22–24], empirical [25] and experimental [26] support for the hypothesis that both harsh (i.e. higher mortality) and unpredictable environments result in higher reproductive effort and lower PI.

Mortality rates and the availability of resources may also interact to influence reproductive decisions [27,28]. For example, women in populations with higher infant mortality, which is often associated with poverty, have earlier first births [29,30]. Therefore, lower socio-economic status and high mortality often predict faster reproductive strategies which are characterized by high fertility, early age of first reproduction, short interbirth intervals (IBI) and low PI [31,32]. Expediting reproduction when expected lifespan is short may be an adaptive strategy that allows individuals to reproduce when mortality is high [26,33].

In addition, because changing conditions can alter the expected returns on investment, individuals are also predicted to adjust their strategies in response to changes in the environment [34]. For example, when mortality is low, higher expected returns on PI may trigger a shift towards more investment in offspring and less in reproduction. Determining precisely how mortality rates affect fertility decisions can be difficult, though. For example, some researchers have argued that parents increase reproduction and shorten IBIs in an effort to compensate for the death of their own children [35,36]. Although this explanation would account for the higher mortality rates often observed in larger families, it presumes that parents have considerable control over their own reproduction, which is a questionable assumption in natural fertility populations [37,38]. Nevertheless, it can be difficult to determine whether this type of ‘replacement’ strategy or competition between siblings over PI is the primary cause of higher mortality rates in larger families [39]. Finally, cultural changes within a given society can also affect trade-offs by shifting the costs between producing and investing in children [40–44].

Analysing the trade-offs between reproduction and investment can be complicated by the genes that are shared by parents and their offspring (e.g. some individuals may experience lower quantity–quality trade-offs because they transfer superior genotypes to offspring) because, like all phenotypic traits, life-history outcomes are the result of an interaction between environments and genes [45,46]. Therefore, distinguishing between the environmental and genetic impacts on traits of close relatives can be difficult. One way to attempt to disentangle these influences, however, is to use heritability estimates which calculate the proportion of a phenotypic trait that is due to genetic variation among individuals in a population [47]. Life-history traits (LHTs), such as lifespan and reproductive success, are closely tied to fitness and are therefore expected to yield lower heritability estimates than traits less related to fitness [45,48,49]. This is because the genetic variance for traits under strong selection should decrease and become fixed in a population. Although heritability estimates are useful for measuring the relative contribution of genes and environments, they can be confounded by shared environments, and fail to provide any information on the source(s) of either the environmental or genetic variance [50,51]. Therefore, any potential genetic effects on life-history trade-offs should be assessed before making inferences about the underlying causes of trade-offs between reproduction and investment in offspring. This is especially true when comparing fitness outcomes between and among close relatives who share both genes and environments [11].

In general, the relationship between fertility, mortality and PI can be difficult to study because of many, often interrelated, factors. For this reason, large datasets which track fertility and mortality across extended periods of time are particularly valuable for analysing the trade-offs parents face between producing and investing in children. Although an analysis of a late-nineteenth century Utah genealogy showed that offspring with more siblings had higher mortality [52], few studies have used genealogical data to closely examine the relationship between mortality, fertility and PI.

This study uses Íslendingabók, a multigenerational genealogy extending back to the founding of Iceland, to explore the impact of reproduction and PI on both parents and children. This database is unique as it is one of the oldest, largest and most comprehensive and accurate population-based genealogies in the world. Iceland is therefore an excellent place to gain insights into the impact of quantity–quality trade-offs in humans.

The aim of this study was to evaluate the relationships and trade-offs between fertility, mortality and PI in Iceland. Specifically, I analysed how the quantity–quality trade-off between fertility and investment in offspring changes in response to environmental conditions. This research examined the relationship of fitness traits between members of the nuclear family to answer three questions. (i) How do members of the immediate family (i.e. siblings and parents) affect each other's lifespan and lifetime reproductive success (LRS)? (ii) Are these effects the result of shared genes or shared environments between family members? (iii) How do these relationships change across time and interact with different environments?

These questions generated five additional predictions that are consistent with life-history theory:

(1) Because each successive child is expected to reduce the total amount of PI available, offspring will incur a fitness cost for each additional sibling they attain.
(2) Because traits related to fitness are expected to have little genetic variance, lifespan and lifetime reproductive similarities among nuclear family members will be the result of a shared environment, rather than shared genes.
(3) Because individuals are expected to accelerate life histories when the risk of extrinsic mortality is higher, lifespan and fertility rates will be negatively correlated.
(4) Because both reproduction and investment in offspring are costs that parents are expected to balance, fertility rates will be negatively correlated with PI while offspring mortality rates will be positively correlated with PI.

## 2. Methods

### 2.1. The database

The analyses are conducted on a proprietary genealogical database, Íslendingabók, owned by deCode Genetics (*n* = 720 000 Icelanders). This is an Icelandic genealogy that extends back to the first inhabitants of the island, circa AD 871. Íslendingabók is unique for several reasons. First, this genealogy is composed of exceptionally accurate and complete life-history data on reproduction and lifespans, owing to excellent record keeping and genetic sequencing. An examination of mitochondrial DNA shows a maternal accuracy rate of 99.3% [53] and the combined non-paternity and laboratory error rate is less than 1.5% [54]. This frequency of extra pair paternity is consistent with rates in other parts of Western Europe over the past 400 years [55]. Although Íslendingabók does not include marriage records, the population is almost exclusively monogamous, especially prior to 1920, with extremely low divorce rates such that less than 5% of individuals in the genealogy have any half-siblings and most of these involved the death of a spouse [56]. Second, the database is a population-based genealogy and, because the Icelandic population has been largely isolated from the rest of Europe, there has been relatively little immigration to or emigration from the island. Finally, because the first national census was conducted in 1703, and Iceland did not undergo the demographic transition until after World War II, this database is unique as it contains over two centuries of meticulously recorded data on a pre-industrial population that did not have access to modern contraceptives [57].

All non-genetic analyses in this study were conducted on individuals who were born between 1700 and 1919 (*n* = 167 280). These dates were chosen to ensure both that the data were reliable (after 1700) and that all individuals had complete life histories. Year of birth, year of death (for individuals who had died) and sex were available for all individuals analysed. Although no data were available on material wealth, occupation or income for any individuals in the genealogy, Iceland is one of the most socio-economically and culturally homogeneous societies in the world and throughout the eighteenth and nineteenth centuries the two major modes of subsistence available to Icelanders were fishing and sheep herding [57–59].

### 2.2. Heritability estimates

The similarities between parents and offspring that result from genetic factors are often referred to as heritability estimates [49–51].^1^ In this study, estimates for the heritability of LHT were obtained by correlating the traits of two types of relatives: (i) parents with their children and (ii) full siblings. Parent–offspring heritability was measured by correlating the mid-parent standardized reproduction or lifespan (e.g. [mother's LRS + father's LRS]/2) with the standardized reproduction or lifespan of their offspring. Full sibling heritability was measured by correlating an individual's standardized LRS (or lifespan) with the standardized LRS (or lifespan) of his/her full siblings. Both full sibling and parent to offspring correlation analyses were carried out with equal weight to nuclear families (table 1). Both these methods are commonly used by researchers to assess the heritability of traits [49,60].

**Table 1.** Pearson correlations for lifespan and lifetime reproductive success (LRS) between parents and offspring and among full siblings. Both longevity and LRS were log_10_-transformed and standardized by decade ((log-transformed value – mean of decade)/s.d. of decade). Analyses were conducted on all individuals born between 1700 and 1919. Full sibling and parent offspring correlation analyses were carried out with equal weight to nuclear families (e.g. a full sibling pair in which the individuals have four full siblings is weighted one-fourth as much as a single full sibling pair).

trait						
	relationship	full siblings (all)	brothers	sisters	opposite sex pairs	partial controlling for birth interval(all)
LRS		0.19 (*p* < 0.01)	0.19 (*p* < 0.01)	0.20 (*p* < 0.01)	0.19 (*p* < 0.01)	0.21 (*p* < 0.01)
		153 084	65 294	58 247	68 413	153 084
lifespan		0.16 (*p* < 0.01)	0.16 (*p* < 0.01)	0.17 (*p* < 0.01)	0.14 (*p* < 0.01)	0.18 (*p* < 0.01)
		153 084	65 294	58 247	68 413	153 084

	relationship	mid-parent: offspring(all)	father–son	mother–daughter	father–daughter	mother–daughter
LRS		0.00 (*p* = 0.08)	0.00 (*p* = 0.77)	−0.01 (*p* < 0.01)	−0.01 (*p* < 0.01)	−0.014 (0.00)
		43 081	21 407	21 812	22 918	20 996
lifespan			0.04 (*p* < 0.01)	0.07 (*p* < 0.01)	0.03 (*p* < 0.01)	0.06 (*p* < 0.01)
			21 407	21 812	22 918	20 996

Heritability estimates based on correlations between relatives depend on the assumption that environmental correlations between individuals are the same across all degrees of relatedness and relationships (e.g. *r* = 1/4 for both aunts and uncles and half-siblings). If closer or different types of relatives have more similar environments, as they often do in humans, the estimates of heritability are biased [47]. Although, on average, half of the genetic material shared by parents, offspring, and full siblings is identical by descent from a common ancestor (*r* = 0.5), the two methods often yield different estimates [47,50]. This is because, in humans, heritability estimates obtained from intra familial regressions are confounded by the likelihood that closely related family members, especially full siblings, share a common environment and receive substantial investment from parents [60–62].^2^

### 2.3. Dependent variables

**Figure 1.**
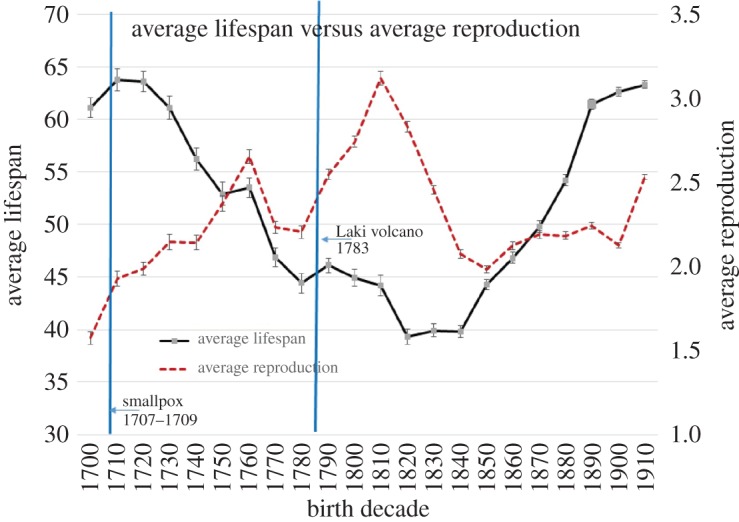
Mean lifespan is negatively correlated with mean lifetime reproductive success (*r* = −0.45, *p* = 0.01) across decades between 1700 and 1919 (e.g. 1700 is average for all individuals born between 1700 and 1709).

Lifespan and LRS were used as dependent variables (hereafter these two traits when combined will be referred to as LHT). LHT fluctuated considerably across time. For example, average fertility rates peak for individuals born between 1810 and 1820 at 3.12 children per individual, but falls to 1.98 just 30 years later, between 1851 and 1860. Mean lifespan also fluctuates considerably over time, peaking for individuals born between 1710 and 1730 at 63.7 years and reaching a low of 39.4 years for individuals born between 1820 and 1840 (figure 1 and electronic supplementary material, table S1A). Therefore, all LHT values were log-transformed and standardized by the decade in which an individual was born ((log-transformed value – mean of decade)/standard deviation of decade). This transformation made the reproduction and lifespan of individuals less dependent on the fluctuations of these traits across birth decades, so that effective comparisons could be made across time. The average of these residuals grouped by family size (number of full siblings) is summarized in table 2. To convert the residuals into numbers that were easier to interpret ‘standardized reproduction’ and ‘standardized lifespan’ (shown in table 2) were both first multiplied by the standard deviation for each category of ‘sibling number’. Next, the mean reproduction or mean lifespan for all individuals born between 1700 and 1919 was added to this value to generate the ‘average reproduction’ and ‘average lifespan’ values in table 2. This was done to provide a reasonable approximation of the average number of years, or children, one loses with the addition of each full sibling.^3^

**Table 2.** Standardized lifespan and LRS are the means of the standardized values ((log-transformed value – mean of decade)/standard deviation of decade). Average reproduction and average lifespan are derived by multiplying the standardized means by the standard deviations for each sibling category and adding them to the mean for all sibling categories across all decades. Analyses were conducted on all individuals born between 1700 and 1919.

sibling number	standardized reproduction	average reproduction	standardized lifespan	average lifespan	*n*
0	0.129 (0.01)	2.71 (0.02)	0.086 (0.00)	52.4 (0.24)	14 736
1	0.079 (0.01)	2.55 (0.02)	0.082 (0.00)	52.2 (0.24)	14 049
2	0.066 (0.01)	2.51 (0.02)	0.079 (0.00)	52.1 (0.22)	16 448
3	0.064 (0.00)	2.50 (0.02)	0.074 (0.00)	52.0 (0.21)	18 560
4	0.052 (0.00)	2.46 (0.02)	0.024 (0.01)	50.5 (0.22)	18 535
5	0.026 (0.01)	2.38 (0.02)	0.007 (0.01)	49.9 (0.23)	17 004
6	0.022 (0.01)	2.37 (0.02)	−0.016 (0.01)	49.2 (0.25)	15 305
7	0.011 (0.01)	2.33 (0.02)	−0.045 (0.01)	48.2 (0.27)	13 317
8	−0.021 (0.01)	2.23 (0.03)	−0.075 (0.01)	47.2 (0.30)	10 472
9	−0.058 (0.01)	2.11 (0.03)	−0.122 (0.01)	45.6 (0.34)	8341
10	−0.036 (0.01)	2.18 (0.03)	−0.140 (0.01)	45.0 (0.38)	6844
>10	−0.077 (0.01)	2.04 (0.02)	−0.173 (0.01)	43.7 (0.26)	14 209

### 2.4. Predictor variables

The primary independent variables used were average reproduction and average lifespan. These were calculated as the means of the raw (unstandardized) LRS and lifespan values for all individuals born within a given decade. I also calculated the average fertility rate of the population per year of life which I have called ‘reproductive effort’.^4^ This was the mean populationwide fertility rate based on the mean lifespan per decade and was a way to determine reproductive rates while controlling for lifespan.

### 2.5. Genetic data

Genetic data were available for 8456 pairs of full siblings who were over the age of 50 and had therefore presumably finished reproducing and 1744 of these pairs were dead (i.e. completed lifespans). Genotypes were obtained by sequencing and comparing individuals on over 300 000 single nucleotide polymorphisms (SNPs). Therefore, extremely accurate relatedness values could be obtained. Specifically, small, but measurable, differences in coefficients of relatedness between full siblings, varying around a mean of 0.5, were used to yield narrow sense heritability estimates^5^ for given traits [61]. The coefficients of relatedness for these individuals were entered into a restricted maximum-likelihood (REML) animal model along with the following control variables: sex, birth year and geographical region. By entering 1s for full siblings and 0s for all other (mostly unrelated) pairs, a ‘family effect’ can be estimated. This model yields different estimates for the effects of genes (SNPs) and the effects of a shared family and is therefore able to distinguish between the effects that result from a common familial environment and those that result from genes [47,51,62].

## 3. Results

### 3.1. Question 1

Overall, there is no detectable relationship between the reproduction of children and their parents (*r* = −0.006, *p* = 0.084, *n* = 43 081) and offspring lifespans (1700–1920) bore only a slight resemblance to those of their parents (*r* = 0.06, *p* < 0.01, *n* = 34 628). Furthermore, there were no notable sex-specific differences for LHT variables between parent–offspring dyads (table 1). Among full siblings, however, there was a positive correlation for both reproduction and lifespan (LRS: *r* = 0.191, *p* < 0.001, *n* = 139 249) (lifespan: *r* = 0.161, *p* < 0.001, *n* = 137 283) (table 1). There were also some interesting sex differences among siblings. Sisters reproduced (*r* = 0.204, *p* < 0.001, *n* = 64 589) and had lifespans (0.167, *p* < 0.001, *n* = 58 247) that were more like their sisters, and brothers reproduced (*r* = 0.198, *p* < 0.001, *n* = 70 636) and had lifespans (*r* = 0.158, *p* < 0.001, *n* = 65 294) that were more like their brothers than opposite sex siblings did for either LRS (*r* = 0.187, *p* < 0.001, *n* = 68 079) or lifespan (*r* = 0.143, *p* < 0.001, *n* = 68 413). Partial correlations controlling for birth intervals between siblings increased both LHT correlations: for LRS (*r* = 0.206, *p* < 0.001, *n* = 139 246) and for lifespan (*r* = 0.178, *p* < 0.001, *n* = 137 280).

### 3.2. Question 1/prediction 1

There was a significant and negative relationship between an individual's number of siblings and their lifespan and LRS. Individuals who had more full siblings, on average, had shorter lifespans and fewer children. Individuals with no siblings lived the longest and had the most children. In sum, each successive sibling reduced LRS by an average of 0.07 children and reduced lifespan by approximately three-fourths of a year (table 2; see Methods for details). An ANOVA test for linearity on both offspring LRS and lifespan showed that sibling number was a significant and negative predictor of both LRS (*r* = −0.052, *p* < 0.001) and lifespan (*r* = −0.082, *p* < 0.001).

### 3.3. Question 2/prediction 2

A REML entering the genetic IBD values for 8456 pairs of full siblings yielded a narrow sense heritability estimate of 0.137 for LRS among full siblings. Adding a family effect to the model and allowing it to compete with these IBD values, however, revealed a family effect of 0.129, s.e. (0.03) and a genetic IBD effect of 0.00, s.e. (0.05). This suggests that the heritability estimate (*h*^2^ = 0.137) for the reproductive relationship between full siblings was based solely on shared family effects among full siblings and was not due to shared genes. Meanwhile, the intraclass correlation of LRS among the 8456 full sibling pairs was *r* = 0.076 (*h*^2^ = 0.152)^6^ reinforcing results from the REML model and also suggesting that the heritability estimate of LRS is lower in modern Iceland. Similar results were obtained for the heritability estimates of lifespan. When both family effects and IBD values were entered for 1744 full sibling pairs, results revealed a family effect of 0.75, s.e. (0.33) and a genetic IBD effect of −0.28, s.e. (0.35). Although these results have high standard errors and are therefore difficult to interpret, they do suggest results similar to those for reproduction among full siblings (e.g. the similarity in the LHTs of full siblings is due to effects from a shared family rather than shared genes). The intraclass correlation of lifespan among these full sibling pairs was *r* = 0.064 (*h*^2^ = 0.152) which both lends support to the REML animal model while also suggesting a lower heritability estimate for lifespan in modern environments (see electronic supplementary material, table S1B). The within family estimates for the heritability of LRS are in line with those obtained previously using Icelandic data [62].

### 3.4. Question 3/prediction 3

Across decades, the mean lifespan of the population was a significant and negative predictor of the mean reproduction of the population (*r* = −0.45, *p* = 0.013; figure 1). A Granger causality test [63] was conducted to determine if there was a predictable time lag between fertility and mortality rates. Results revealed that average fertility rate was a significant predictor of average lifespan (i.e. mortality) one decade later (*F* = 10.85, *p* = 0.004) and two decades later (*F* = 4.48, *p* = 0.02).

### 3.5. Question 3/prediction 4

**Figure 2.**
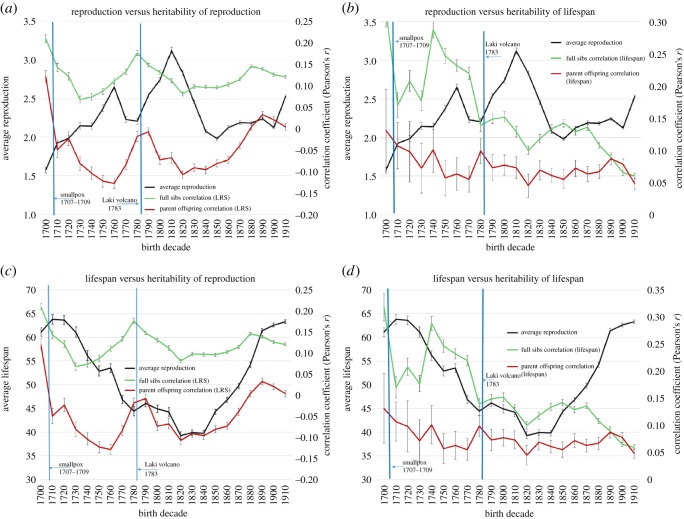
(*a*) Mean reproduction is negatively correlated with the heritability estimates (double the Pearson correlations) of the lifetime reproductive success (LRS) among full siblings (*r* = −0.61, *p* < 0.001) and between parents and offspring (*r* = −0.44, *p* = 0.04) across decades (1700–1919). Heritability estimates for LRS between parents and offspring and among full siblings are also positively correlated (*r* = 0.73, *p* < 0.001). (*b*) Mean reproduction is negatively correlated with the heritability estimates (double the Pearson correlations) for the lifespan among full siblings (*r* = −0.35, *p* = 0.10) and between parents and offspring (*r* = −0.54, *p* = 0.009) across decades (1700–1919). Heritability estimates for lifespan between parents and offspring and among full siblings are also positively correlated (*r* = 0.48, *p* = 0.02). (*c*) Mean lifespan is positively correlated with the heritability estimates (double the Pearson correlations) for LRS among full siblings (*r* = 0.29, *p* = 0.17) and between parents and offspring (*r* = 0.51, *p* = 0.016) across decades (1700–1919). Heritability estimates between parents and offspring and among full siblings are also positively correlated (*r* = 0.73, *p* < 0.001). (*d*) Mean lifespan is positively correlated with the heritability estimates (double the Pearson correlations) of lifespan among full siblings (*r* = 0.16, *p* = 0.47) and between parents and offspring (*r* = 0.46, *p* = 0.03) across decades (1700–1919). Heritability estimates between parents and offspring and among full siblings are also positively correlated (*r* = 0.48, *p* = 0.02).

The heritability estimates (*h*^2^ = Pearson's *r *× 2) among full siblings and between parents and offspring fluctuate considerably across decades but are strongly and positively correlated with each other for both LHTs: LRS (*r* = 0.73, *p* < 0.001) and lifespan (*r* = 0.71, *p* < 0.001). Average reproduction, meanwhile, is negatively correlated with heritability estimates obtained both from full siblings: LRS (*r* = −0.605, *p* = 0.001) and lifespan (*r* = −0.45, *p* = 0.035), and from parent and offspring: LRS (*r* = −0.44, *p* = 0.041) and lifespan (*r* = −0.54, *p* = 0.009; figure 2*a*,*b*). Correlations between ‘reproductive effort’ (see Methods) and all four heritability estimates yield similar results: with full siblings, LRS (*r* = −0.30, *p* = 0.168) and lifespans (*r* = −0.33, *p* = 0.15) and with parents and offspring: LRS (*r* = −0.52, *p* = 0.012) and lifespans (*r* = −0.54, *p* = 0.009) (see electronic supplementary materials, figure S1A). In contrast, average lifespans are positively correlated with the heritability estimates obtained both from full siblings: LRS (*r* = 0.29, *p* = 0.17) and lifespan (*r* = 0.16, *p* = 0.47), and from parents and offspring: LRS (*r* = 0.51, *p* = 0.016) and lifespan (*r* = 0.46, *p* = 0.03; figure 2*c*,*d*).

To help determine whether the heritability estimates derived from the LRS correlations among full siblings were the result of a shared household or were due to other environmental factors, I also looked at average heritability estimates for LRS for first cousins (*r* = 0.06, *p* < 0.001, *n* = 671 849). There was a positive correlation between the heritability estimates obtained for full siblings and those obtained from first cousins *r* = 0.45 (*p* = 0.04) but no detectable relationship between average reproduction and the LRS estimates among first cousins *r* = 0.08 (*p* = 0.73) (see electronic supplementary material, figure S1B).

## 4. Discussion

### 4.1. Heritability estimates measure the non-genetic impact of parents

This study showed that LRS and lifespans (LHTs) of full siblings are positively correlated suggesting that parents affect the LHTs of their offspring. The lack of any strong overall correlation between the LRS or lifespans of parents and their offspring, however, indicates that it is the environment shared by siblings, rather than genes, that drives this relationship. If genes were the sole determinant of the similarities among full siblings, then we would expect the relationship between the LHT of parents and offspring to be the same as it is among full siblings. This is because both groups (full siblings and parents and offspring) have the same average genetic coefficient of relatedness (*r* = 0.5). The genetic analysis of SNPs among full siblings supports this interpretation and indicates an extremely low effect of genes on the LHTs of individuals. Together these findings suggest that the environments provided by parents have had important impacts on the LHT of offspring and support the hypothesis that resources and the impact of PI^7^ have had important influences on both reproduction and lifespan.

These results are consistent with empirical evidence [39,44,46] and evolutionary theory [5–12]. LHTs that are closely tied to fitness, such as LRS and lifespan, usually yield lower heritability estimates than traits less tied to fitness, such as height or eye colour [46]. Therefore, these observations provide empirical support for Fisher's fundamental theorem of natural selection [45] which states that, for a population in equilibrium, there is no additive genetic variance in total fitness because the genetic variance for traits under strong selection is expected to be depleted [64]. Results from the genetic and statistical analysis of full sibling pairs in this study lend support to this conclusion.

There are important differences between the environments we share with full siblings and those we share with parents. First, only full siblings share parents; and second only full siblings share a household before they are sexually mature. Therefore, shared environments and resources are expected to impact full siblings and parents in different ways. Despite differences in these shared environments, the observation that the relationship among full siblings is positively correlated with the relationship between parents and offspring suggests that they are linked and may be measuring similar things (figure 2*a*–*d*).^8^ The estimates obtained from the correlations between parents and offspring may be interpreted as the quantity–quality trade-off faced by parents but may also provide insight into levels of PI. When the LHT correlations between parents and offspring are lower, full siblings are costly and PI may therefore also be low, but when parent–offspring correlations are higher, additional full siblings do not negatively affect one's fitness, and we may expect that levels of PI are higher. Meanwhile the heritability estimates obtained from the correlations among full siblings can also be interpreted as a proxy for the effect that PI has on offspring fitness.^9^ When siblings reproduce alike and have similar lifespans, the effect of parents tends to be high but when these traits are less similar, their effect may be lower. This interpretation is reasonable as long as we assume that part of the non-genetic similarity among full siblings is due to their parents.^10^

### 4.2. The relationship between fertility, mortality and parental investment

This study demonstrates a negative correlation between fertility and mortality. In decades when average lifespans are short, average reproduction is high and during decades where average lifespans are long, average reproduction is low (figure 1). Results of a Granger test of causality suggest that these changes in fertility precede the changes in mortality. This result contrasts with the predictions of many economic models of the demographic transition [65], the expectations of many human behavioural ecologists [27,34] and several historical datasets which show the opposite pattern (i.e. changes in mortality precede changes in fertility) [58]. Evidence from nineteenth century England and France [65] and, more recently, from several less developed countries [66], however, reveals a pattern that is similar to the one shown here. Why might changes in fertility precede changes in mortality? First, higher fertility rates have been associated with lower parental survivorship, particularly for mothers, and this may result in lower offspring survival [28,29]. Lower average family sizes may also increase the amount of parental care that is available per child which could, in turn, reduce offspring mortality rates.

The heritability estimates may also provide insights into what is driving changes in fertility rates. Average reproduction is high, not only when mortality rates are high (figure 1), but also when both LHT heritability estimates are low (figure 2*a*,*b*). This relationship suggests that parents have a stronger influence on their children (high heritability estimates) when fertility rates are low. Overall, the negative relationship between fertility and mortality rates (figure 1), combined with the negative relationship between reproduction and the overall impact of parents on offspring fitness (figure 2*a*,*b*), suggest that parents balance reproductive effort with investment. By reducing reproduction and increasing investment, they can positively affect the fitness of their offspring. The observation that mean lifespans are positively related to all the LHT heritability estimates provides additional evidence that additional investment may reduce offspring mortality (figure 2*c*,*d*). Finally, the negative relationship of both mortality rates and heritability estimates with reproductive effort (i.e. fertility rates/average lifespans) indicates that these associations are not simply the result of individuals dying before they have had the chance to reproduce.

In an attempt to determine whether the environments shared by full siblings were the result of shared geographical region (e.g. shared villages) or the result of common households (e.g. PI), I examined the heritability of LRS among first cousins. While first cousins are likely to live in the same geographical area, they do not share parents or households. Although the heritability estimates for LRS derived from first cousins are positively correlated with the estimates obtained from full siblings, there is no relationship between the estimates obtained from cousins and overall fertility rates (see electronic supplementary material, figure S1B). These results support the hypothesis that fertility rates are linked to shared parents rather than shared villages. Overall, the interactions between fertility, mortality and the heritability estimates indicate that parents face a trade-off between investment in reproductive effort and investment in offspring. Lower fertility rates appear to be related to higher PI or higher competition for parental resources, which in turn may reduce child mortality.

### 4.3. The quality–quantity trade-off responds to environmental conditions

These relationships also seem to change over time in response to environmental conditions. To gain insights into the ways in which fertility and investment decisions respond to the environment, we can observe the impact of two catastrophic events in Icelandic history on heritability estimates and fertility rates. Although Icelandic history is replete with chronic suffering, extreme conditions and disasters, two stand out as extraordinary [67]. The first of these was the smallpox epidemic of 1707–1709, which is projected to have killed 26% of the population [68,69]. The second was the Laki volcanic eruption of 1783 during which 25% of the population and more than 50% of the livestock are estimated to have died [70]. These events are both associated with rapid declines in population density and harsh environmental conditions. One interpretation of these patterns is that when conditions worsen, individuals respond by reducing PI (i.e. full sibling and parent offspring correlations decline) and increasing reproductive effort (figure 2*a,b*). This view is also consistent with the hypothesis that trade-offs between reproduction and investment depend on expected returns on investment (e.g. individuals respond to poor conditions and less predictability by increasing reproduction and reducing investment in offspring) [19,20,71].

### 4.4. Evolutionary theory and implications for understanding the demographic transition

The demographic transition is characterized by the replacement of high fertility, high mortality and low PI by low fertility, low mortality and high PI [72]. Evolutionary explanations of this process often focus on the idea that in economically developed countries, the costs of producing and raising children are higher [73,74]. These costs, whether real or merely perceived, may contribute to lower fertility rates in populations that either have undergone or are undergoing socio-economic development. This reasoning has been used to explain why wealth is often associated with higher offspring status but lower reproductive success in post-demographic transition populations [73–78].^11^ If across human evolution child survivorship, rather than total number of births, has been the primary determinant of fitness [79], and resources (i.e. status) have been closely aligned with offspring survival [80–82], then the perceived risk of offspring mortality will have an important impact on the expected fitness returns on investment. Human behavioural ecologists, for example, have suggested that an evolved psychology which ties social status to fitness helped drive the rapid fertility declines and increases in PI that characterize the demographic transition [73,81]. This is fuelled by expectations of higher returns on PI whenever child mortality falls.

Although the demographic transition does not occur in Iceland until the middle of the twentieth century, these data suggest that the same relationships between fertility, mortality and investment in offspring that propel the demographic transition are at work throughout the eighteenth and nineteenth centuries. In decades in which PI is presumed to be low (i.e. both the parent offspring and full sibling correlations are low), fertility rates are high (figure 2*a*,*b*). In other words, falling fertility is coincident with rising investment, and this ultimately leads to lower mortality. This lends support to the hypothesis that fertility rates may be sensitive to the expected fitness returns per unit of investment.

## 5. Conclusion

There was a quantity–quality trade-off between the reproduction of parents and the fitness of their children. Each additional sibling reduced an individual's expected lifespan by almost one year and their LRS by 0.07 children.^12^ In addition, the genetic effects of parents on offspring are extremely low suggesting that LHT similarities among members of the nuclear family are likely due to the effects of shared environments and PI. The observation that there is no detectable historic relationship between fertility rates and the LRS of first cousins lends further support to this interpretation. Together, these results indicate that increasing reproduction negatively impacts offspring fitness by reducing the resources or investment available for each offspring.

There are also several lines of evidence suggesting that environmental conditions have affected this trade-off. First, when conditions are good and mortality rates are low, fertility rates are also low. Second, populationwide fertility rates are negatively correlated with the LHT heritability estimates, indicating that when populationwide fertility rates are high, the impact of parents is low. Third, low mortality rates are associated with high heritability estimates, suggesting either that PI reduces childhood mortality or that parents invest more in offspring when mortality rates are low. Finally, acute mortality shocks are associated with increased fertility rates and a coincident reduction in the impact of parents, which implies that harsh conditions trigger an increase in reproductive effort and a decrease in PI per offspring.

There are reasons to be cautious when interpreting these results, however. For example, the negative relationship between mortality and fertility may be caused by parents increasing reproduction in an effort to replace their own dead children [83] rather than by a facultative response to environmental conditions. This interpretation would also explain why individuals with more siblings have shorter lives and lower fertility (table 2; i.e. because larger families have higher child mortality). Although it can be difficult to determine whether parents are responding to general environmental conditions or if they are responding specifically to the deaths of their own children, the results presented in table 1 and figure 2*a*–*d* provide some reasons to doubt the latter. First, controlling for the birth intervals between siblings does not reduce the LHT correlations (table 1). If parents were simply replacing their own children, especially infants, then we would expect to see shorter birth intervals in larger families. Instead the partial correlations increase, which indicates that the observed similarities between the lifespans and LRS of full siblings are not due to differences in birth intervals. Second, the observation that individuals live and reproduce more like their siblings than their parents suggests that PI has an important impact on the fitness of their children. Finally, the fact that the LHT relationships both among full siblings and between parents and offspring are negatively correlated with populationwide fertility rates but positively correlated with mean lifespans, indicates that parents are adjusting investment strategies in response to communitywide mortality rates, rather than only to the deaths of their own children. Nevertheless, it may also be the case that some of the relationships between the LHTs of close relatives result simply from competition between siblings irrespective of parental resources or investment.

Overall, these results provide evidence that individuals face a trade-off between producing and investing in children and that changing environmental conditions may cause parents to adjust these strategies. These results suggest that a flexible balance between investment in offspring and reproductive effort may have played an important role in human evolution.

## Supplementary Material

Figure A: Mean reproductive effort (average reproduction/ average lifespan) is negatively correlated with mean lifespan across decades between 1700 and 1919 (e.g. 1700 is average for all individuals born between 1700 and 1709).

## Supplementary Material

Figure B: Mean reproduction is not correlated with the heritability of lifetime reproductive success amongst first cousins across decades (1700–1919). Heritability estimates between first cousins are 8 X the Pearson correlations shown here. All correlations were carried out with equal weight to extended families.

## Supplementary Material

Table A: Summary data used to create all figures (figure 1, 2a, 2b, 2c and 2d, and supplementary materials figures A and figure B.

## Supplementary Material

Table B: Narrow sense heritability estimates (h2) generated from a comparison of full sibling pairs across approximately 300,000 single nucleotide polymorphisms. All 8,456 full sibling pairs who were measured for lifetime reproductive success were over the age of 50 and all 1,744 who were measured for lifespan were dead.

## Footnotes

1 The narrow-sense heritability of a phenotype (*h*^2^) is the fraction of phenotypic variance that can be described by an additive model over the set of SNPs that are functionally related to the phenotype (i.e. the causal SNPs).

2 Assuming random mating, and no dominance, maternal or interaction effects and negligible covariance between phenotypic values and environmental deviation, narrow sense heritability [49] for parents and offspring and full siblings can be estimated as twice the Pearson's correlation: *h*^2^ = 2*r*.

3 This transformation was necessary to control for fluctuating lifespans and fertility across decades. For example, it is the negative relationship between one's number of siblings (i.e. larger family sizes) and one's fitness may be spurious, because mortality was higher (e.g. shorter lifespans) in decades when families were smaller (mean reproduction was lower). Converting these residuals back into a real number just provides a rough idea of how many years, or children, are lost (on average) for each sibling gained.

4 Although reproductive effort is typically used to assess the amount of energy an individual organism devotes towards reproduction, I have used it here as a population wide estimate to determine fertility rates that are independent of mortality rates (lifespans) across decades.

5 Narrow sense heritability estimates (*h*^2^) are the proportion of genetic variation that is due to additive genetic values.

6 To determine *h*^2^ from correlation among sibling and between parents and offspring one must divide the correlation coefficient by the coefficient of relatedness, or in the case of parents and siblings this can be done by simply doubling the Pearson correlation.

7 Resources refer to parental resources distributed among offspring, but the environments shared by full siblings may consist of several factors other than direct PI and resource distribution which social and cultural influences such as fertility preferences.

8 The correlation among full siblings may be higher than between parents and offspring owing to a shared environment (e.g. PI), but could also result from dominance, epistatic or maternal effects. Meanwhile, the SNP data among full siblings are only measuring additive genetic variance which does not include epistatic, dominance or maternal effects. I suggest that the shared environment amongst siblings is the most likely reason for the higher correlation, because the other genetic effects (epistasis and dominance) are less likely to be so volatile across decades (figure 2*a*–*d*). Maternal effects, on the other hand, may be an important cause of the difference, but these effects are subsumed under PI. Finally, trait similarity between relatives is expected to be mostly driven by additive genetic variance (narrow sense heritability estimates *h*^2^) while response to selection also depends on additive genetic variance [60].

9 Although we do not know whether actual PI was high or low, the impact of PI on offspring fitness may be estimated from the correlations among full siblings.

10 Throughout the rest of the Discussion section, the correlation among the reproduction or lifespans of full siblings and the parent–offspring correlations are interpreted broadly as a proxy for the impact of parents on offspring fitness, and sometimes more specifically as the effect that PI has on offspring fitness.

11 In modern environments, however, where offspring survival is almost guaranteed and few individuals lack the resources to reproduce, there is often a mismatch where high status does not necessarily lead high fitness outcomes.

12 Although a reduction of almost a year in lifespan may suggest a reasonably impactful effect size, it may be argued that a reduction of 0.07 children per sibling is not particularly strong. This may be especially true because average family sizes in Iceland were relatively small (e.g. less than 5). However, owing to the high number and complexity of the factors (e.g. resources, PI and countless others) that contribute to LRS, I would argue that an effect size of 0.07 children per sibling is actually quite high. This may be particularly true if, as suggested by researchers and historians, wealth and family size in eighteenth and nineteenth century Iceland are positively correlated.

## Data accessibility

The dataset on which this analysis was conducted is available on request from deCode Genetics: Agnar Helgason, email: agnar.helgason@decode.is. The supporting data are available on Dryad: http://dx.doi.org/10.5061/dryad.q3nn7.
