# Supplementary figures and images for: Parents face quantity–quality trade-offs between reproduction and investment in offspring in Iceland
Source: R Soc Open Sci. 2016 May 18;3(5):160087. doi: 10.1098/rsos.160087 (PMC4892449; doi:10.1098/rsos.160087)

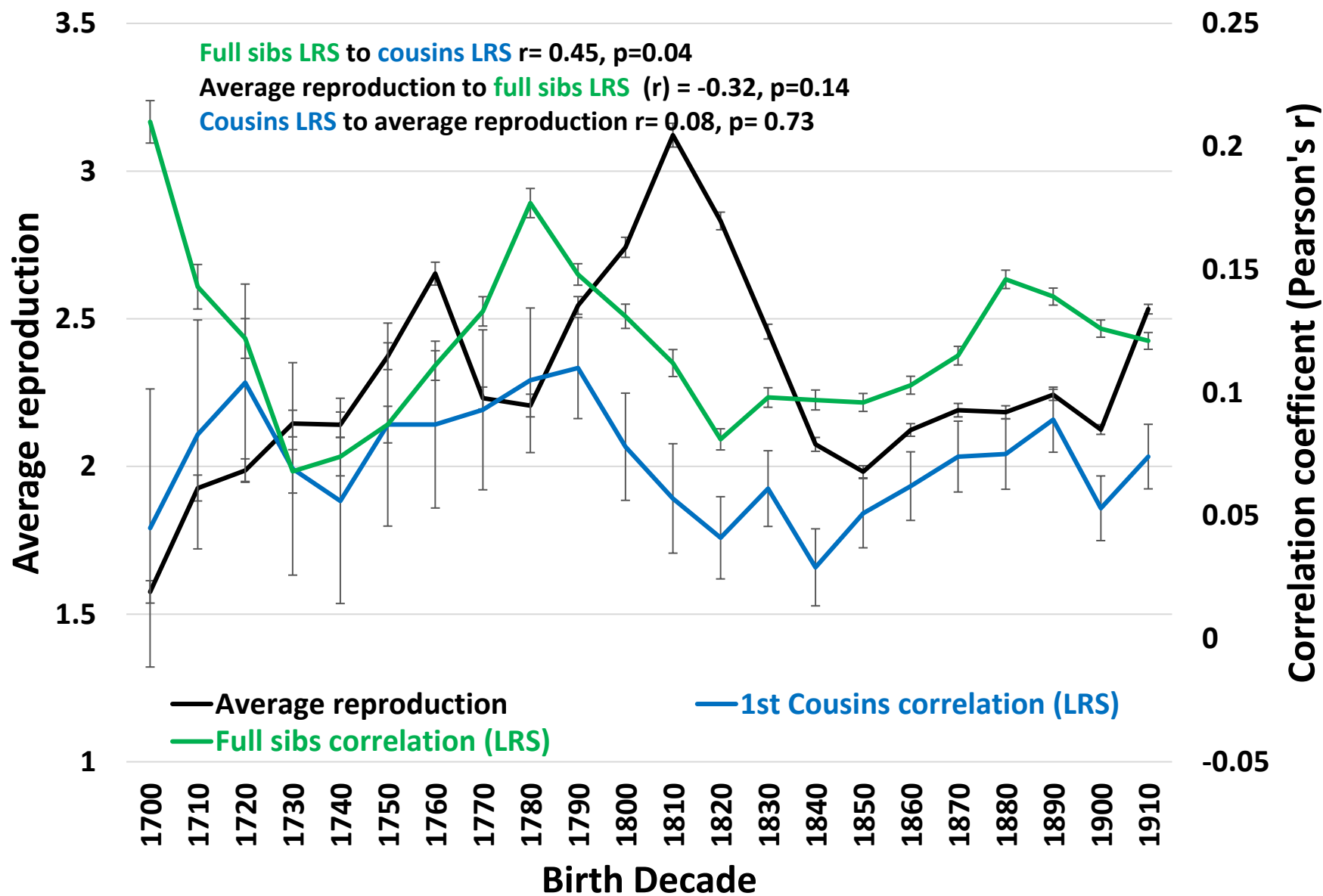

Supplement: Figure A: Mean reproductive effort (average reproduction/ average lifespan) is negatively correlated with mean lifespan across decades between 1700 and 1919 (e.g. 1700 is average for all individuals born between 1700 and 1709). [file rsos160087supp1.pdf]

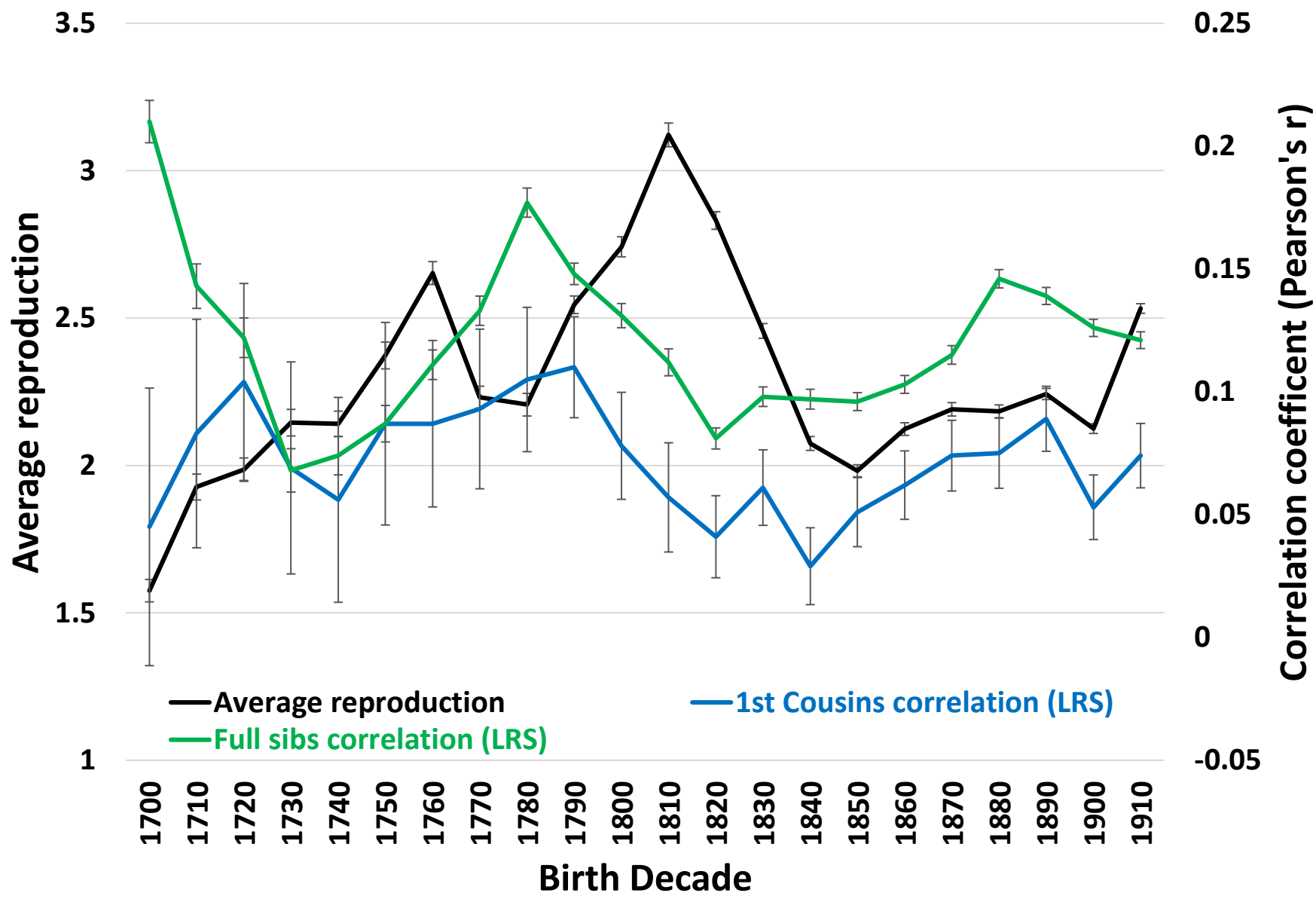

Supplement: Figure B: Mean reproduction is not correlated with the heritability of lifetime reproductive success amongst first cousins across decades (1700–1919). Heritability estimates between first cousins are 8 X the Pearson correlations shown here. All correlations were carried out with equal weight to exte [file rsos160087supp2.pdf]
