# Supplementary material for: Parents face quantity–quality trade-offs between reproduction and investment in offspring in Iceland
Source: R Soc Open Sci. 2016 May 18;3(5):160087. doi: 10.1098/rsos.160087 (PMC4892449; doi:10.1098/rsos.160087)
Supplement: Table A: Summary data used to create all figures (figure 1, 2a, 2b, 2c and 2d, and supplementary materials figures A and figure B. [file rsos160087supp3.pdf]

Birth deca	Full sibs correlation (LRS)	N	S.E.	Full sibs cc	N	S.E.	Parent offs
1700	0.21	3286	8.62E-03	0.318	322	0.0256	0.121
1710	0.143	2946	9.05E-03	0.17	644	0.0195	-0.049
1720	0.122	3785	8.06E-03	0.208	938	0.0159	-0.023
1730	0.068	3221	8.82E-03	0.177	1062	0.0152	-0.081
1740	0.074	4047	7.83E-03	0.288	1370	0.0126	-0.104
1750	0.087	4440	7.44E-03	0.247	1596	0.012	-0.123
1760	0.111	6717	6.02E-03	0.232	2585	9.50E-03	-0.128
1770	0.133	6886	5.97E-03	0.22	2551	9.61E-03	-0.084
1780	0.177	7061	5.93E-03	0.139	2486	0.01	-0.018
1790	0.148	13229	4.37E-03	0.149	4515	7.43E-03	-7.00E-03
1800	0.131	10323	4.93E-03	0.152	3746	8.14E-03	-0.073
1810	0.112	8424	5.49E-03	0.129	3370	8.64E-03	-0.068
1820	0.081	13762	4.29E-03	0.1	5194	7.01E-03	-0.107
1830	0.098	15982	3.98E-03	0.118	5882	6.56E-03	-0.091
1840	0.097	15527	4.05E-03	0.134	5858	6.55E-03	-0.096
1850	0.096	19124	3.64E-03	0.142	8028	5.58E-03	-0.081
1860	0.103	19137	3.64E-03	0.129	8711	5.38E-03	-0.073
1870	0.115	17587	3.78E-03	0.136	9430	5.16E-03	-0.039
1880	0.146	17892	3.74E-03	0.108	10684	4.88E-03	4.00E-03
1890	0.139	20999	3.47E-03	0.089	14815	4.16E-03	0.033
1900	0.126	20886	3.50E-03	0.065	16344	3.97E-03	0.021
1910	0.121	21932	3.41E-03	0.06	14909	4.16E-03	4.00E-03

N	S.E.	Parent offs	N	S.E.	1st Cousin	N	S.E.	Average re
1390	0.0135	0.131	70	0.0638	0.045	1200	0.056489	1.5753
789	0.0181	0.107	102	0.0361	0.083	1753	0.046504	1.9269
1058	0.0157	0.098	115	0.0472	0.104	2340	0.040088	1.9859
892	0.0169	0.072	170	0.0389	0.069	2044	0.043157	2.1452
1248	0.0144	0.101	210	0.0351	0.056	2197	0.041694	2.1412
1362	0.0136	0.057	329	0.0279	0.087	2226	0.041237	2.3732
2035	0.0112	0.063	397	0.0256	0.087	3296	0.033887	2.6527
2051	0.0112	0.055	655	0.0199	0.093	3577	0.032493	2.2316
1790	0.0121	0.099	690	0.0194	0.105	4355	0.029376	2.2062
3056	9.23E-03	0.073	641	0.02	0.11	8872	0.020558	2.5451
2308	0.0105	0.077	1063	0.0156	0.078	8004	0.021776	2.7418
1802	0.012	0.073	907	0.0168	0.057	7724	0.022231	3.1215
2812	9.47E-03	0.045	797	0.018	0.041	13726	0.016702	2.8311
3322	8.76E-03	0.069	1190	0.0148	0.061	16118	0.015381	2.4565
2995	9.24E-03	0.061	2057	0.0112	0.029	15667	0.015646	2.0747
3697	8.33E-03	0.055	1204	0.0147	0.051	19332	0.01406	1.9819
3937	8.09E-03	0.072	1607	0.0127	0.062	19641	0.013932	2.1235
3962	8.10E-03	0.063	1836	0.0118	0.074	18334	0.014396	2.1905
4263	7.81E-03	0.067	2085	0.0111	0.075	18622	0.014283	2.1836
4994	7.20E-03	0.087	2419	0.0103	0.089	21613	0.013227	2.2425
4613	7.51E-03	0.078	3308	8.81E-03	0.053	22125	0.01314	2.125
4872	7.31E-03	0.048	3628	8.42E-03	0.074	22101	0.013112	2.5326

Average reproduction standard error	Average lifespan	Average Lifepan standard error
0.03795659	61.1	0.936837055
0.043806825	63.8	1.057579263
0.039685385	63.6	0.95653498
0.045117912	61.1	1.087341688
0.043084224	56.2	1.038329787
0.045383796	52.9	1.09374948
0.038791153	53.5	0.934866791
0.037184749	46.9	0.896152458
0.038399177	44.4	0.925420176
0.029962979	46.1	0.722107805
0.034036444	44.9	0.8202783
0.040248008	44.2	0.969976997
0.0298823	39.3	0.720163424
0.025008107	39.9	0.602695373
0.023499328	39.8	0.566333815
0.020526038	44.3	0.494677524
0.02138912	46.8	0.515477788
0.022844376	49.8	0.550549459
0.021846592	54.2	0.526502865
0.018918806	61.4	0.455943222
0.016424702	62.6	0.395835311
0.016230115	63.3	0.391145769
