# Supplementary material for: Parents face quantity–quality trade-offs between reproduction and investment in offspring in Iceland
Source: R Soc Open Sci. 2016 May 18;3(5):160087. doi: 10.1098/rsos.160087 (PMC4892449; doi:10.1098/rsos.160087)
Supplement: Table B: Narrow sense heritability estimates (h2) generated from a comparison of full sibling pairs across approximately 300,000 single nucleotide polymorphisms. All 8,456 full sibling pairs who were measured for lifetime reproductive success were over the age of 50 and all 1,744 who were measured f [file rsos160087supp4.pdf]

Full siblings	narrow sense heritability estimate genetic effect (S.E.)	narrow sense heritability estimate family effect (S.E.)	N
Lifetime Reproduction	0.00 (0.05)	0.129 (0.03)	8456
Lifespan	-0.28 (0.35)	0.75 (0.33)	1744
Height	0.71 (0.25)	0.003 (0.13)	6159
